# Proprietaries

**Published:** 1904-12

**Authors:** 


### PROPRIETARIES.

During the meeting of the American Medical Association held at Atlantic City last June, representatives of the State medical journals conceived and promulgated the idea that mutual interests would be better served, a bond of sympathy and good will created between themselves, and the profession at large benefited by an association termed "The American Association of State Medical Journals."

One of their number, whose bump of wisdom was more normally developed than that of some of those present, succeeded in having final action on the project deferred until the meeting of the A. M. A. at Portland, Ore., next year.

The points of special interest in their declaration of principles are:

(1) No journal of this association shall accept an advertisement of a medicine which is not ethical, and "ethical" shall mean that the product advertised must have published with it not only the names of its constituent parts, but also the amount of such constituents, so that a definite dosage can be determined. Further, such product must not be advertised in the secular press, to the laity.

(2) If a product is marketed under a copyrighted name, the manufacturer shall furnish with it the proper chemical name, and if not patented, then also the process of manufacture.

(3) All advertisements not covered by the above paragraphs, or which contain extravagant or improbable claims, shall be submitted to the executive committee for approval before they can be accepted.

If the representatives present had any desire or wish for anything further they did not express it, and it is fair to presume that they do not want anything more. Were an excessive or inordinate desire for gain present they might have demanded a share of stock in each company who patronized them or levied on a portion of their patrons' receipts—pabulum for the "kitty" as it were, but as nothing of this sort was done, they can not be accused of having had the slightest mercenary or other improper motives when their declaration of principles was formulated.

If they are ever put into actual operation the "bond of sympathy" ought to be ordered exceptionally strong, as it is not difficult to foresee that the demands made upon it will be unusually heavy.

There is probably no question but that State medical journals, at least the more influential ones, can be conducted without a loss, without a page of advertising matter, as members of the State associations are in a measure morally bound to pay their subscriptions, if, in fact, the State association does not set aside a dollar or two from the annual dues of the members as a reserve for the expenses of their official organ, but the question of profit had better not be discussed.

It is also true that many independent medical journals could be issued regularly from month to month from the amounts received from subscribers, but it is safe to assume that medical editors and publishers "live not by glory alone," and do, undoubtedly, depend upon the commercial side for their maintenance and profit to a considerable extent.

Notwithstanding the enormous circulation of some of the great daily papers of our large cities, but a small proportion of the excellent service we all enjoy is due to the amount received from subscriptions, and were it not for the general advertiser, the modern newspaper with its foreign news department as completely served as its domestic, its elegant press work and fine illustrations, brilliant and lucid editorials written by men whose salaries run into the five figures, would dwindle to a mere job sheet, more despised than read.

As this project is scheduled to lie over until next annual meeting of the A. M. A., there is ample time for a thorough consideration of the subject, and although no prophet, nor the son of a prophet, we venture the prophecy that its consummation will never be accomplished—unless suicide is contemplated, and that the executive committee who are delegated to pass upon the propriety of proposed advertisement will not be compelled to shorten their office hours nor curtail their outside work in order to perform their duties.

Medicinal preparations of a proprietary nature possess a distinctive character as marked as that of individuals, due to the process of manufacture and fixed composition. Many of the better-known preparations would not suffer by the publication of their exact formulæ, as, for example, when complicated laboratory machinery is required for their perfect manufacture, but this is not true of many well-known preparations whose nature is that of a simple compound.

It is, however, also true that if the exact formulæ of certain proprietary remedies were known and taken to a number of skilled pharmacists for compounding the results would differ in nearly every instance.

It is not to be supposed that a preparation can always be duplicated providing the formula is known, but such a procedure would encourage substitution to an alarming extent, and in this fact lies the greatest danger. While substitution is probably not practiced as generally as some writers would lead one to believe, especially by the retail druggist or individual prescriptions, there is another form of substitution which, while in a sense not deceptive nor fraudulent, is nevertheless an imposition upon the original manufacturer and a source of positive financial loss to him.

A common example is the preparation Essence of Pepsin. Every druggist can make it, and practically all do make it, all perhaps using the standard formula, yet, write ten prescriptions and compare results! Compound Syrup of Hypophosphites is another example; this preparation can be found on the shelf of every drug-store in the country, yet in appearance, taste and action all differ, and in many instances materially.

This form of substitution is found in the drug-stores presumed to be of the higher grade, the store located on the principal street and the most prominent corner. The proprietor has a certain percentage more brains than his uptown or downtown competitor; he has the ability to develop his one time drug-store into a department store with a drug department; he is patronized by the elite, and the high-priced doctors send their prescriptions to him for compounding.

Does this druggist substitute? Not much! He has sufficient intelligence to see beyond the extra dime or quarter profit he might make by using a cheap substitute for a high-priced proprietary; he argues that he charges good prices for his prescriptions and can afford to buy all the high-priced chemicals and compounds that the doctor may write for, but his keenly developed mental equipment soon reaches the conclusion that there is a shorter road to wealth than by the prescription, via proprietary remedy, route.

By the aid of his knowledge of chemistry and pharmacy, and a few timely hints by the editor of his drug journal, he soon perfects an elegant imitation of a certain proprietary, which, strange to relate, often possesses more virtue and curative power than the original (?).

He now calls upon Dr. So and So and the others who have favored him with their business, thereby conceding evidence of their confidence in him and mutely acknowledging their belief in his superior ability, and in a few well-chosen words convinces the doctor that it is foolish to pay one dollar an ounce, or one dollar a pint for a remedy that can be duplicated for less than half that amount, as he supposes. The doctor's attention is called to the fact that he, as an intelligent and educated physician, can, of course, readily see that nothing is gained by adhering to the old and genuine preparation, and the result is, he prescribes the druggists' imitation product more or less afterward. By continual efforts in this direction, worthy of a nobler purpose, doctors are constantly imposed upon through a want of a proper knowledge of the facts, but who believe the statements repeatedly made by the interested parties.

That harm frequently results from lack of precaution on the part of the physician there can be no doubt, yet, the remedies prescribed are not always absolutely indicated and in many cases the expected results are not obtained, even when the genuine remedies are dispensed, but when this is the case under the best possible conditions, what can one expect from the use of imitations that are known to be such?

Apart from therapeutic views, it is unfair, venal, dishonest. Persons who further their own interests by appropriating the discoveries of others and who confiscate the products of the brains of their superiors, are conducting their business along lines which the self-respecting and conscientious physician can not follow with profit to himself nor advantage to his patient.

Anything done that will make substitution easy, or which will enable the skilled but dishonest pharmacist to become a party to the deception of physicians, can not but be looked upon as a step directly unwise and in direct opposition to true progress and meritorious advancement.

The outcome of the plans proposed by the A. of S. M. J. will be watched with interest.—*Editorial Albright's Office Practitioner.*

